# Supplementary material for: Abundance does not predict extinction risk in the fossil record of marine plankton
Source: Commun Biol. 2023 May 22;6:554. doi: 10.1038/s42003-023-04871-6 (PMC10203123; doi:10.1038/s42003-023-04871-6)
Supplement: Supplementary file 2 — Supplementary Figures [file 42003_2023_4871_MOESM2_ESM.pdf]

Supplementary Figures

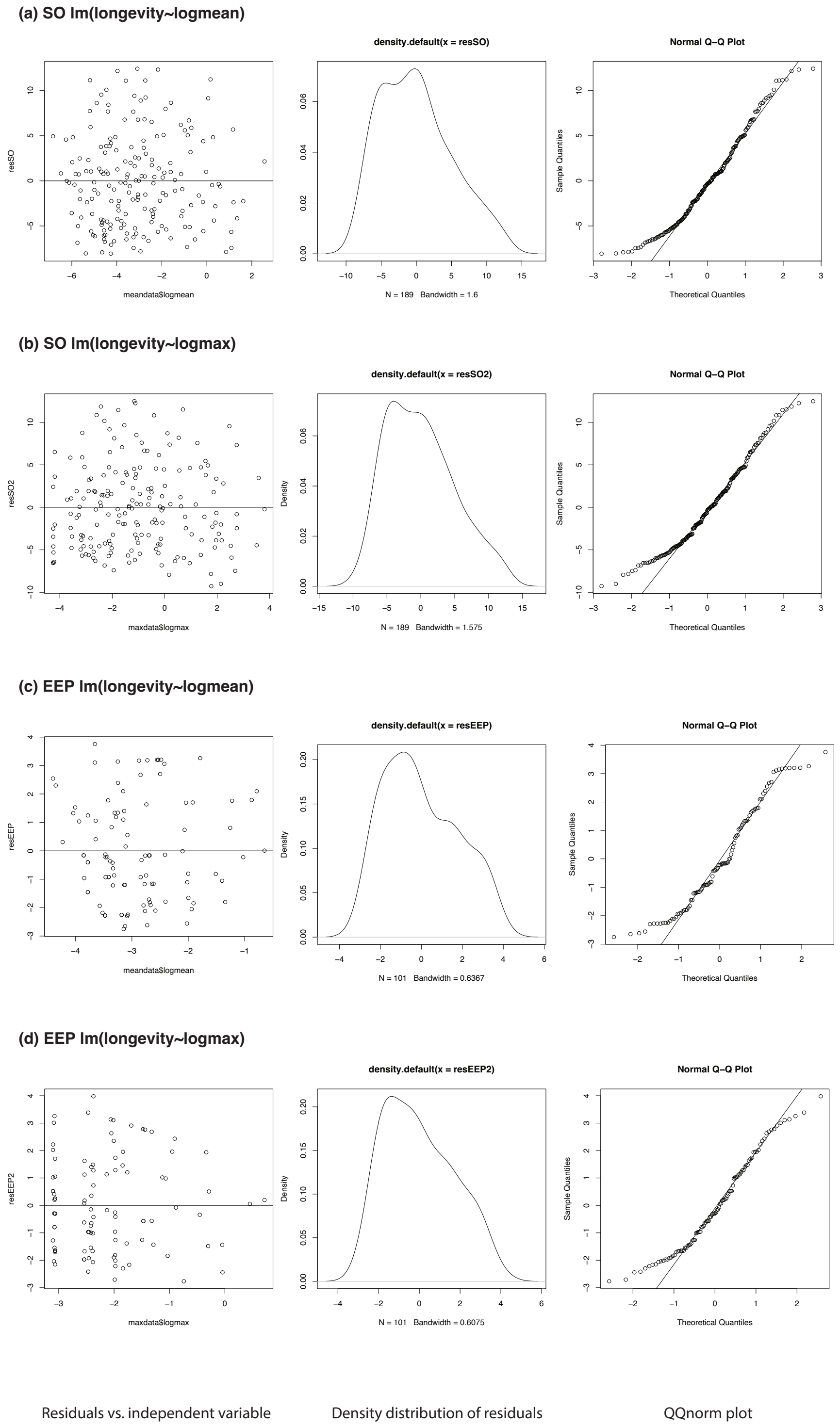

Supplementary Figure 1. Residuals plots for all linear regression models illustrated in Figure 2. These regression models include: Southern Ocean (SO) average relative abundance vs. longevity **(a)**; SO maximum relative abundance vs. longevity **(b)**; eastern equatorial Pacific

(EEP) average relative abundance vs. longevity **(c)**; and EEP maximum relative abundance vs. longevity **(d)**. Plots in the left column show the independent variable in each regression analysis (x) versus the model residuals (y). These suggest that the choice of linear model is appropriate (no evidence of a non-linear relationship). The center column consists of density plots indicating the distribution of model residuals. These show that residuals are roughly normally distributed. The right column gives the QQnorm plots, also an indication that residuals are normal.

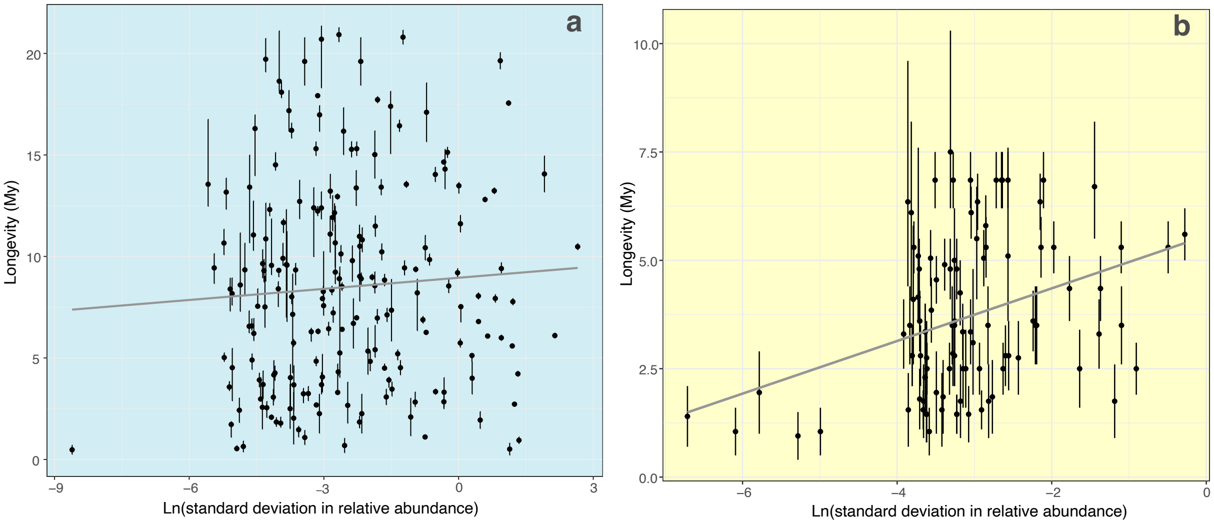

y = 0.1823ln(x) + 8.9501
R-squared: 0.0046
p-value= 0.3515

y = 0.6ln(x) + 5.5676
R-squared: 0.1197
p-value = 0.0004

Supplementary Figure 2. Linear regression model results for standard deviation in relative abundance as a predictor for radiolarian species longevity in the Southern Ocean (SO) **(a)** and eastern equatorial Pacific (EEP) **(b)**. The SO analysis shows no significant relationship between standard deviation in abundance and longevity. The EEP analysis suggests a very weak positive relationship between standard deviation in abundance and longevity, but this pattern is driven by five outlier species and does not reflect an overall trend in the data (see Results in main text). N=189 species in **(a)** and n=101 species in **(b).** Error bars on longevity are designated for each species based on its average gap size between occurrences (see Methods and Supplementary Data 1).

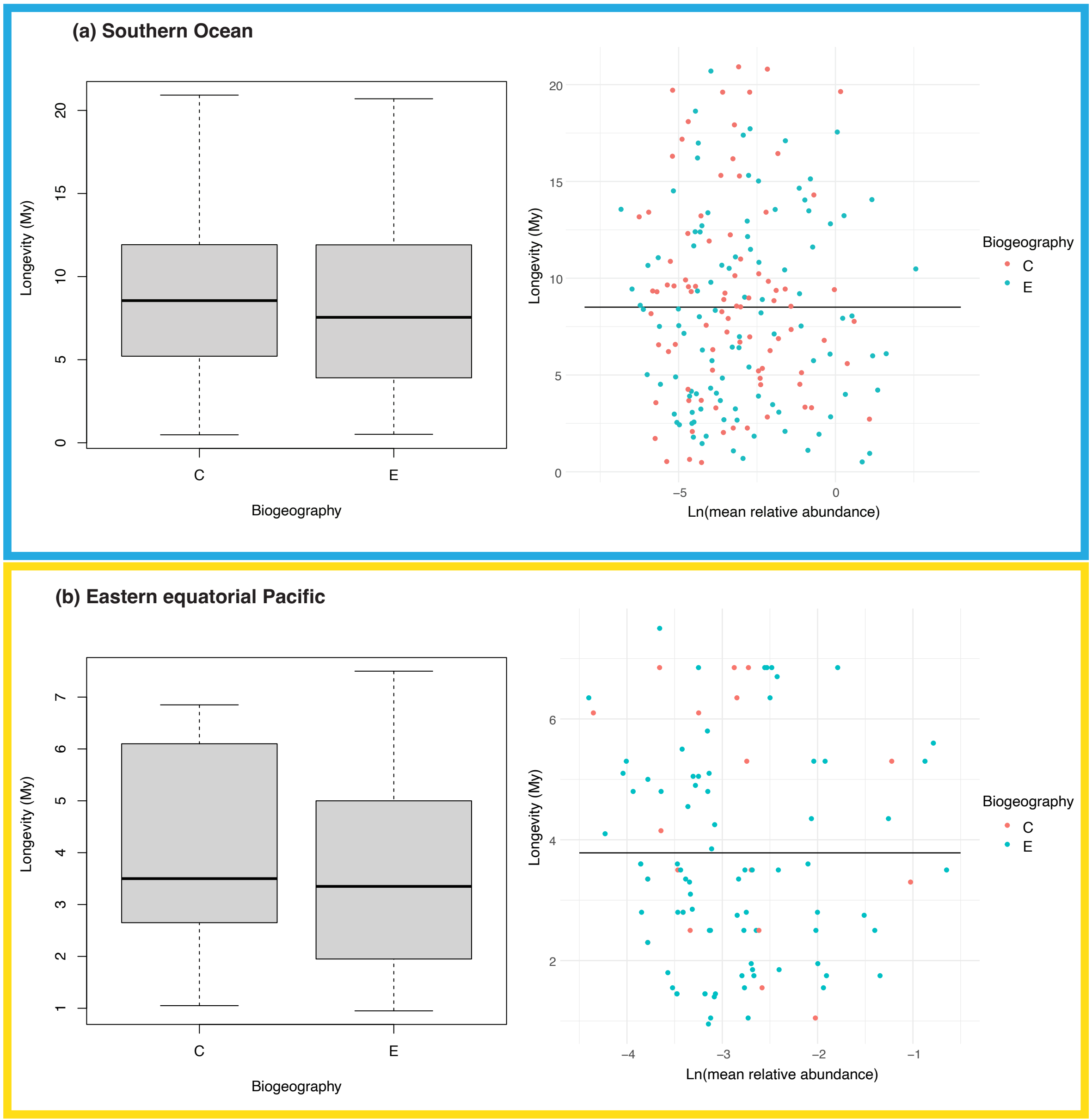

Supplementary Figure 3. Analysis of species biogeographic range as a predictor of longevity in the Southern Ocean (SO) **(a)** and eastern equatorial Pacific (EEP) **(b)** datasets. C= cosmopolitan; E=endemic. Boxplots show the median, interquartile range, maximum, and minimum values for longevity of the cosmopolitan and endemic radiolarian species in each region. N=189 species in **(a)** and n=101 species in **(b)**. The plots on the right show the natural log of mean relative abundance values (x) vs. longevity (y), with a linear mixed effects model plotted in black. The linear mixed effects models combine biogeographic classification and abundance as the predictor of longevity, but the regressions show no relationship in either oceanographic region. Cosmopolitan species are indicated with red points and endemic species with blue.
