# Supplementary material for: Abundance does not predict extinction risk in the fossil record of marine plankton
Source: Commun Biol. 2023 May 22;6:554. doi: 10.1038/s42003-023-04871-6 (PMC10203123; doi:10.1038/s42003-023-04871-6)
Supplement: Supplementary file 3 — Description of Additional Supplementary Files [file 42003_2023_4871_MOESM3_ESM.pdf]

Description of Additional Supplementary Files

File name: Supplementary Data 1
Description: Raw radiolarian abundance data underlying the analyses in this article. The Southern Ocean (SO) dataset is presented in the first sheet (Table 1) and the eastern equatorial Pacific (EEP) dataset is given in the second sheet (Table 2). The number of individuals of each species observed in each sample are provided, along with the total number of species-level specimens counted in each sample. Occurrences in red text were omitted from analyses due to suspected reworking or misidentifications (see Methods). First and last occurrence dates were estimated based on average gap size in the species' occurrence record (see Methods); uncertainty is given in the "Error bar" column and illustrated with highlighted cells (yellow= origination date range, red=extinction date range). "Max first occurrence date" and "max last occurrence date" refer to the oldest dates estimated by our method in millions of years (Ma). "Min first occurrence date" and "min last occurrence date" refer to the youngest dates estimated by our method in millions of years (Ma). "First occurrence date (midpoint)" and "Last occurrence date (midpoint)" are the dates used to estimate the longevity values used in our analyses, given in the column "Longevity (midpoint)" in millions of years. "Longevity (max)" and "Longevity (min)" represent the upper and lower bounds on longevity estimates (in millions of years), respectively. Mean relative abundance, maximum relative abundance, and standard deviation (SD) in relative abundance are listed for each species across its stratigraphic range. The "Group" column refers to the taxonomic order of each species; C=Collodaria, N=Nassellaria, S=Spumellaria. The "Biogeography" column lists the biogeographic category of each species, based on whether it was present in only the SO or EEP ("endemic") or both regions ("cosmopolitan"); E=endemic, C=cosmopolitan.
