# Supplementary material for: Abundance does not predict extinction risk in the fossil record of marine plankton
Source: Commun Biol. 2023 May 22;6:554. doi: 10.1038/s42003-023-04871-6 (PMC10203123; doi:10.1038/s42003-023-04871-6)
Supplement: Supplementary file 5 — Reporting Summary [file 42003_2023_4871_MOESM5_ESM.pdf]

nature portfolio

Corresponding author(s): Sarah Trubovitz & Johan Renaudie

Last updated by author(s): Apr 14, 2023

Reporting Summary

Nature Portfolio wishes to improve the reproducibility of the work that we publish. This form provides structure for consistency and transparency in reporting. For further information on Nature Portfolio policies, see our Editorial Policies and the Editorial Policy Checklist.

Statistics

For all statistical analyses, confirm that the following items are present in the figure legend, table legend, main text, or Methods section.

n/a	Confirmed	
☐	☒	The exact sample size (*n*) for each experimental group/condition, given as a discrete number and unit of measurement
☐	☒	A statement on whether measurements were taken from distinct samples or whether the same sample was measured repeatedly
☐	☒	The statistical test(s) used AND whether they are one- or two-sided<br>*Only common tests should be described solely by name; describe more complex techniques in the Methods section.*
☒	☐	A description of all covariates tested
☐	☒	A description of any assumptions or corrections, such as tests of normality and adjustment for multiple comparisons
☐	☒	A full description of the statistical parameters including central tendency (e.g. means) or other basic estimates (e.g. regression coefficient) AND variation (e.g. standard deviation) or associated estimates of uncertainty (e.g. confidence intervals)
☐	☒	For null hypothesis testing, the test statistic (e.g. *F*, *t*, *r*) with confidence intervals, effect sizes, degrees of freedom and *P* value noted<br>*Give P values as exact values whenever suitable.*
☒	☐	For Bayesian analysis, information on the choice of priors and Markov chain Monte Carlo settings
☒	☐	For hierarchical and complex designs, identification of the appropriate level for tests and full reporting of outcomes
☒	☐	Estimates of effect sizes (e.g. Cohen's *d*, Pearson's *r*), indicating how they were calculated

Our web collection on statistics for biologists contains articles on many of the points above.

Software and code

Policy information about availability of computer code

Data collection: All data used in this study were previously collected and published in Trubovitz et al (2020) and Renaudie & Lazarus (2013), and are available at: https://doi.org/10.5281/zenodo.4014322. No additional software or code was used for data collection.

Data analysis: Built-in functions in R v. 3.6.0 and the R package lme4 were used for data analyses. The open-source R packages ggplot2 and ggOceanMaps were used for illustration.

For manuscripts utilizing custom algorithms or software that are central to the research but not yet described in published literature, software must be made available to editors and reviewers. We strongly encourage code deposition in a community repository (e.g. GitHub). See the Nature Portfolio guidelines for submitting code & software for further information.

Data

Policy information about availability of data

All manuscripts must include a data availability statement. This statement should provide the following information, where applicable:

- Accession codes, unique identifiers, or web links for publicly available datasets
- A description of any restrictions on data availability
- For clinical datasets or third party data, please ensure that the statement adheres to our policy

All data analyzed during this study are available in the Zenodo repository (https://doi.org/10.5281/zenodo.4014322). The specific subset of these data we used in the present study are provided as a supplementary dataset along with this article (Supplementary Data 1).

Human research participants

Policy information about studies involving human research participants and Sex and Gender in Research.

Reporting on sex and gender	N/A
Population characteristics	N/A
Recruitment	N/A
Ethics oversight	N/A

Note that full information on the approval of the study protocol must also be provided in the manuscript.

Field-specific reporting

Please select the one below that is the best fit for your research. If you are not sure, read the appropriate sections before making your selection.

☐ Life sciences ☐ Behavioural & social sciences ☒ Ecological, evolutionary & environmental sciences

For a reference copy of the document with all sections, see nature.com/documents/nr-reporting-summary-flat.pdf

Ecological, evolutionary & environmental sciences study design

All studies must disclose on these points even when the disclosure is negative.

Study description	This study utilized Neogene and Quaternary fossil polycystine radiolarian occurrence and abundance data obtained from deep sea sediments representing the eastern equatorial Pacific and the Southern Ocean. Our goal was to determine whether abundance, higher taxon identity, or biogeographic range size are related to species longevity. The data we analyzed were originally published in Trubovitz et al (2020), and comprise a radiolarian biodiversity census from 10.3 Ma–Recent in the eastern equatorial Pacific, and 22.65–0.04 Ma in the Southern Ocean. For each oceanographic region, we identified the individual species with complete biostratigraphic ranges represented in the dataset (details in “Methods”). Average, maximum, and standard deviation in relative abundance for these species were calculated from the raw biodiversity census data. Regression analyses were then used to test for a relationship between any metric of abundance (independent variable) and species longevity (dependent variable). The potential control of higher taxonomy (Order-level) on species longevity was tested using analyses of variance (ANOVA). Biogeographic range category (cosmopolitan versus endemic) was also considered as a predictor for species longevity and tested using linear mixed effects models and ANOVA. Results were interpreted in the context of ecological neutral theory and niche theory, and implications for modern biodiversity conversation are discussed.
Research sample	Samples are comprised of fossilized polycystine radiolarian specimens obtained from deep sea sediments, and processed to include siliceous material in the >45 µm size fraction. This size fraction was used to account for the minimum size of polycystine radiolarian species known from previous research. Prior work indicates that the vast majority of original polycystine radiolarian diversity is expected to be present in the fossil record; thus, each sample is considered broadly representative of the polycystine radiolarian community at a given time. Only samples with age-depth models rated as “Good” or better were included in our analyses, to ensure the accuracy of sample age estimates.
Sampling strategy	No new sampling was conducted as part of this study. Samples were enumerated by J. Renaudie in Renaudie and Lazarus (2013) and S. Trubovitz in Trubovitz et al (2020) to obtain occurrence and abundance data for all polycystine radiolarian species within each time slice. During data collection, sample completeness was determined by visually examining collection curve flattening. To verify consistent completeness among samples, established coverage metrics were calculated to indicate completeness values ranging from 93-99% (see Methods in Trubovitz et al 2020). For the eastern equatorial Pacific dataset, approximately 5,200 specimens were identified per sample, with an average of ~2,500 of these specimens identified to the species level: a total of 35,311 species-level observations across 14 samples. For the Southern Ocean dataset, the average number of specimens identified to the species level per sample is 7,190: a total of 697,396 species-level observations across 97 samples.
Data collection	The dataset published by Trubovitz et al (2020) was filtered by Trubovitz in 2021 to include only the species with first and last occurrences represented in each study interval (study intervals include the last 10.3 million years for the eastern equatorial Pacific, and the last 22.65 million years for the Southern Ocean). Any species with a documented occurrence in the two oldest or youngest samples in either region was removed, as well as any species that had an average occurrence gap size indicating its range likely extends outside the study interval (i.e., species that could potentially be extant, or have originated prior to 10.3 Ma in the eastern equatorial Pacific or prior to 22.65 Ma in the Southern Ocean). Details on this vetting procedure are given in “Methods.” The average and maximum relative abundance for species with fully represented ranges was calculated from the complete species dataset in Trubovitz et al (2020). Stratigraphic range duration paired with relative abundance metrics for 101 eastern equatorial Pacific species, and 189 Southern Ocean species served as the dataset for all analyses conducted in this study.
Timing and spatial scale	The Southern Ocean dataset spans 22.65–0.04 Ma, and includes 97 samples from International Ocean Discovery Program (IODP) Sites 689, 690, 744, 747, 748, 751, and 1138, which are circumpolarly distributed around the Southern Ocean. The eastern equatorial Pacific dataset contains 14 samples spanning 10.3 – 0 Ma, all of which were obtained from IODP Site U1337. The latter

	data were compared to previous tropical Pacific radiolarian diversity records (all longitudes, ≤ 20° N/S) in the global microfossil database, Neptune Sandbox Berlin, to confirm that data from Site U1337 are representative of tropical radiolarian biodiversity over the last 10 million years (see full comparative analysis in Trubovitz et al 2020).
Data exclusions	Some taxon occurrences were excluded if there was reason to believe they were misidentifications or reworked specimens, based on careful reexamination of specimen photographs and the ranges of known biostratigraphic marker taxa (these occurrences are marked with red text in Supplementary Data 1). Out of 203,229 occurrence observations of the species with full ranges represented in the Southern Ocean dataset, a total of 102 observations were excluded (0.05%). In the eastern equatorial Pacific dataset, 1 out of 967 taxon occurrence observations of species with full ranges was excluded (0.1%).
Reproducibility	We did not attempt to reproduce this study. However, robust sample standardization and taxon range filtering techniques were used to verify our results.
Randomization	The specimens in each sample were randomly-settled from solution onto glass cover slips during slide-making (see Methods in Trubovitz et al 2020). This procedure ensures that each slide displays a random subset and even distribution of specimens from the sample.
Blinding	Blinding was not relevant to this study, since the fossil specimens were not subjected to experimental treatments.

Did the study involve field work? ☐ Yes ☒ No

Reporting for specific materials, systems and methods

We require information from authors about some types of materials, experimental systems and methods used in many studies. Here, indicate whether each material, system or method listed is relevant to your study. If you are not sure if a list item applies to your research, read the appropriate section before selecting a response.

Materials & experimental systems

n/a	Involved in the study
☒	☐ Antibodies
☒	☐ Eukaryotic cell lines
☐	☒ Palaeontology and archaeology
☒	☐ Animals and other organisms
☒	☐ Clinical data
☒	☐ Dual use research of concern

Methods

n/a	Involved in the study
☒	☐ ChIP-seq
☒	☐ Flow cytometry
☒	☐ MRI-based neuroimaging

Palaeontology and Archaeology

Specimen provenance	No new specimens were obtained for this study. All material was originally acquired from the International Ocean Discovery Program (IODP). Details of specimen provenance are provided in Renaudie and Lazarus (2013) and Trubovitz et al (2020).
Specimen deposition	The Southern Ocean microfossil specimens referred to in this study have been deposited by Renaudie and Lazarus at the Museum für Naturkunde in Berlin. The eastern equatorial Pacific specimens are scheduled to be deposited at the Museum für Naturkunde in early 2023.
Dating methods	No new dates are provided; ages are from published age-depth models for the specific deep sea sediment cores used.

☒ Tick this box to confirm that the raw and calibrated dates are available in the paper or in Supplementary Information.

Ethics oversight	No ethical approval or guidance was required as our study did not involve human or other living subjects.

Note that full information on the approval of the study protocol must also be provided in the manuscript.
